# Spatiotemporal dynamics of epidemiology diseases: mobility based risk and short-term prediction modeling of COVID-19

**DOI:** 10.3389/fpubh.2024.1359167

**Published:** 2024-07-03

**Authors:** Melissa Silva, Cláudia M. Viana, Iuria Betco, Paulo Nogueira, Rita Roquette, Jorge Rocha

**Affiliations:** ^1^Associated Laboratory TERRA, Institute of Geography and Spatial Planning, University of Lisbon, Lisbon, Portugal; ^2^Associated Laboratory TERRA, Nursing Research, Innovation and Development Centre of Lisbon (CIDNUR), Nursing School of Lisbon, Lisbon, Portugal; ^3^Instituto de Saúde Ambiental (ISAMB), Faculdade de Medicina, Universidade de Lisboa, Lisbon, Portugal; ^4^Escola Nacional de Saúde Pública, ENSP, Centro de Investigação em Saúde Pública, CISP, Comprehensive Health Research Center, CHRC, Universidade NOVA de Lisboa, Lisbon, Portugal; ^5^NOVA IMS Information Management School, NOVA University of Lisbon, Lisbon, Portugal

**Keywords:** mobility, risk, non-linearity, ergodic, forecasting, simple exponential smoothing

## Abstract

Nowadays, epidemiological modeling is applied to a wide range of diseases, communicable and non-communicable, namely AIDS, Ebola, influenza, Dengue, Malaria, Zika. More recently, in the context of the last pandemic declared by the World Health Organization (WHO), several studies applied these models to SARS-CoV-2. Despite the increasing number of researches using spatial analysis, some constraints persist that prevent more complex modeling such as capturing local epidemiological dynamics or capturing the real patterns and dynamics. For example, the unavailability of: (i) epidemiological information such as the frequency with which it is made available; (ii) sociodemographic and environmental factors (e.g., population density and population mobility) at a finer scale which influence the evolution patterns of infectious diseases; or (iii) the number of cases information that is also very dependent on the degree of testing performed, often with severe territorial disparities and influenced by context factors. Moreover, the delay in case reporting and the lack of quality control in epidemiological information is responsible for biases in the data that lead to many results obtained being subject to the ecological fallacy, making it difficult to identify causal relationships. Other important methodological limitations are the control of spatiotemporal dependence, management of non-linearity, ergodicy, among others, which can impute inconsistencies to the results. In addition to these issues, social contact, is still difficult to quantify in order to be incorporated into modeling processes. This study aims to explore a modeling framework that can overcome some of these modeling methodological limitations to allow more accurate modeling of epidemiological diseases. Based on Geographic Information Systems (GIS) and spatial analysis, our model is developed to identify group of municipalities where population density (vulnerability) has a stronger relationship with incidence (hazard) and commuting movements (exposure). Specifically, our framework shows how to operate a model over data with no clear trend or seasonal pattern which is suitable for a short-term predicting (i.e., forecasting) of cases based on few determinants. Our tested models provide a good alternative for when explanatory data is few and the time component is not available, once they have shown a good fit and good short-term forecast ability.

## 1 Introduction

The periodic re-emergence of infectious diseases such as tuberculosis, diphtheria, malaria and cholera (1), as well as the emergence of new pathogens, such as Severe Acute Respiratory Syndrome (SARS) or Human immunodeficiency virus (HIV) (2), reinforce the need for epidemiological science endowed with increasingly complex models (3). The growing technological sophistication has contributed to the development of models that allow the incorporation of health, economic, social and behavioral contexts, among others, in the disease modeling process (4). Among these advances is the growing importance of the spatial dimension in epidemiological modeling to capture the spatiotemporal dynamics of infectious processes (5). Epidemiological modeling allows the temporal and dynamic study of the evolution of diseases and the creation of scenarios related to their transmission, constituting knowledge for the control of the same. Nowadays, epidemiological modeling is applied to a wide range of diseases, communicable and non-communicable, namely AIDS, Ebola, influenza, cancer, Dengue, Malaria, Zika (6). More recently, in the context of the last pandemic declared by the World Health Organization (WHO), several studies applied these models to SARS-CoV-2 (7–9).

SARS-CoV-2 was the most recent world’s leading public health problem declared as a pandemic on March 12, 2020, by the WHO. From the pandemic’s start until January 22, 2022, there have been 364,191,494 confirmed cases and 5,631,457 deaths worldwide in 117 countries (10–12). Despite the increasing number of researches using spatial analysis applied to SARS-CoV-2, some constraints persist that prevent more complex modeling such as capturing local epidemiological dynamics or capturing the real patterns and dynamics (13). For example the unavailability of: (i) epidemiological information such as the frequency with which it is made available; (ii) sociodemographic and environmental factors (e.g., population density and population mobility) at a finer scale which influence the evolution patterns of infectious diseases (14, 15); or (iii) the number of cases information that is also very dependent on the degree of testing performed, often with severe territorial disparities and influenced by context factors (16). Moreover, the delay in case reporting and the lack of quality control in epidemiological information is responsible for biases in the data that lead to many results obtained being subject to the ecological fallacy (15), making it difficult to identify causal relationships. Other important methodological limitations are mentioned in (17), such as the control of spatiotemporal dependence, management of non-linearity, ergodicy, among others, which can impute inconsistencies to the results. In addition to these issues, social contact, the real responsible for the transmission of SARS-CoV-2, is still difficult to quantify in order to be incorporated into modeling processes.

Recent reports on communicable diseases demonstrate that inequalities constitute conditions for the transmission of infectious diseases which supports the notion that pandemics are social rather than health care problems (18, 19). For example, human concentration in cities and urban areas is a factor that leads to the existence of a suitable environment for the transmission of infectious agents. The behavioral of individuals is critical for the emergence, dispersion and containment of diseases (20). The patterns of population movement and the way in which they occur in the territory (within the parish, between municipalities, by personal car or public transport) are characteristics that potentially can influence the spread of infectious diseases (21). Population movements are essential for social contact to occur, so mobility, in the context of infectious diseases, becomes a determining factor (22). Particularly, urbanization rate can act as a determinant for the occurrence of infectious outbreaks (23) and may play different roles in the context of disease transmission (24), nevertheless is the population density that has been identified as a significant factor for the proliferation of infectious diseases (25).

Information on population mobility patterns during the pandemic can constitute a proxy for social contact, however, the availability of this information in the current patterns of periodicity, aggregation and scales of analysis has some shortcomings that are not allow their use for large-scale predictive epidemiological modeling (13, 26). In this context, network diffusion model becomes particularly relevant because social contacts are transmission channels for the dissemination of any phenomenon (27). With great suitability for transmitting ideas on social networks (28), the network diffusion model is also representative of the transmission of diseases through social contact in physical proximity being an important model in infectious contexts (29, 30).

Accordingly, this study aims to explore a modeling framework that can overcome some of the above-methodological limitations to allow more accurate modeling of epidemiological diseases. Based on Geographic Information Systems (GIS) and spatial analysis, our model is developed to identify group of municipalities where population density (vulnerability) has a stronger relationship with incidence (hazard) and commuting movements (exposure). Specifically, our framework shows how to operate a model over data with no clear trend or seasonal pattern (31) which is suitable for a short-term predicting (i.e., forecasting) of cases base on few determinants. This study introduces a novel modeling framework designed to overcome these limitations. Utilizing Geographic Information Systems (GIS) and spatial analysis, our model identifies clusters of municipalities where population density, disease incidence, and commuting patterns are closely interlinked. Unlike existing models that require extensive data and temporal trends, our approach is optimized for short-term forecasting based on a limited set of determinants. Preliminary tests indicate a strong model fit and reliable short-term predictions. This framework offers a robust alternative for scenarios lacking comprehensive data and time components, thereby contributing to more accurate and adaptable epidemiological modeling. The model has significant implications for public health policy, particularly in addressing local epidemiological dynamics and territorial disparities.

## 2 Materials and methods

### 2.1 Study area

**Figure 1:**
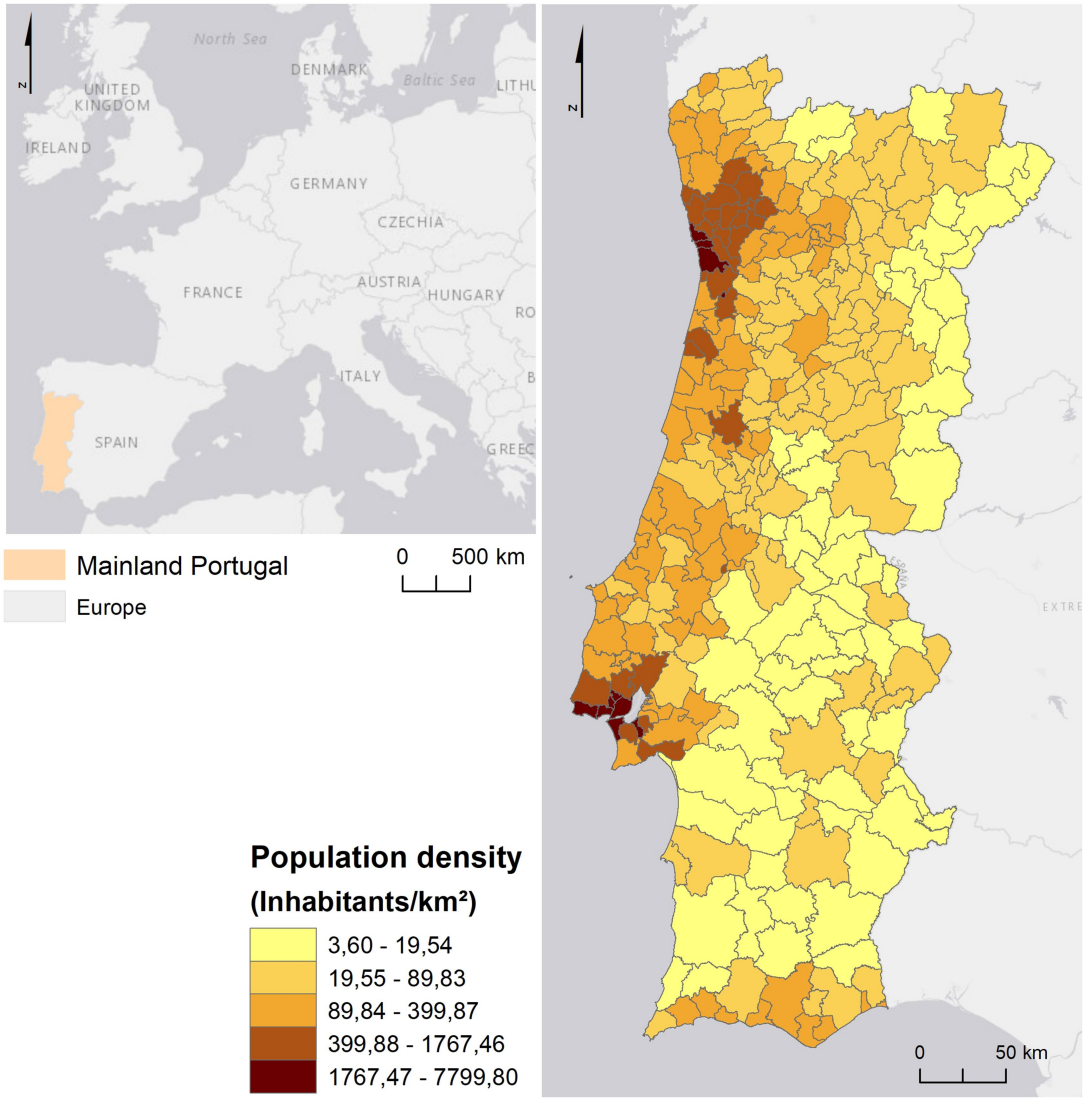
Context of Mainland Portugal in Europe.

Portugal is located at the southwestern tip of Europe. It has a land border with Spain to the North and East and a maritime border to the South and West with the Atlantic Ocean (Figure 1). The Portuguese territory includes two autonomous regions: the archipelagos of the Madeira and Azores (32) (which were not considered in the study). Portugal has a total area of 92,225 km2, of which 89,102 km2 correspond to mainland Portugal (33).

The resident population in mainland Portugal was 9,860,175 inhabitants in 2021 (34). The territory is characterized by a coastal/inland dichotomy regarding population distribution (35), since, in general, the coast has a higher population density than the interior, as shown in Figure 1. However, the population distribution along the Portuguese coast also presents a heterogeneous pattern, since it is evident the contrast between the metropolitan areas of Lisbon and Porto in relation to the remaining territory of mainland Portugal, in which most municipalities have more than 399.88 individuals per km2.

**Figure 2:**
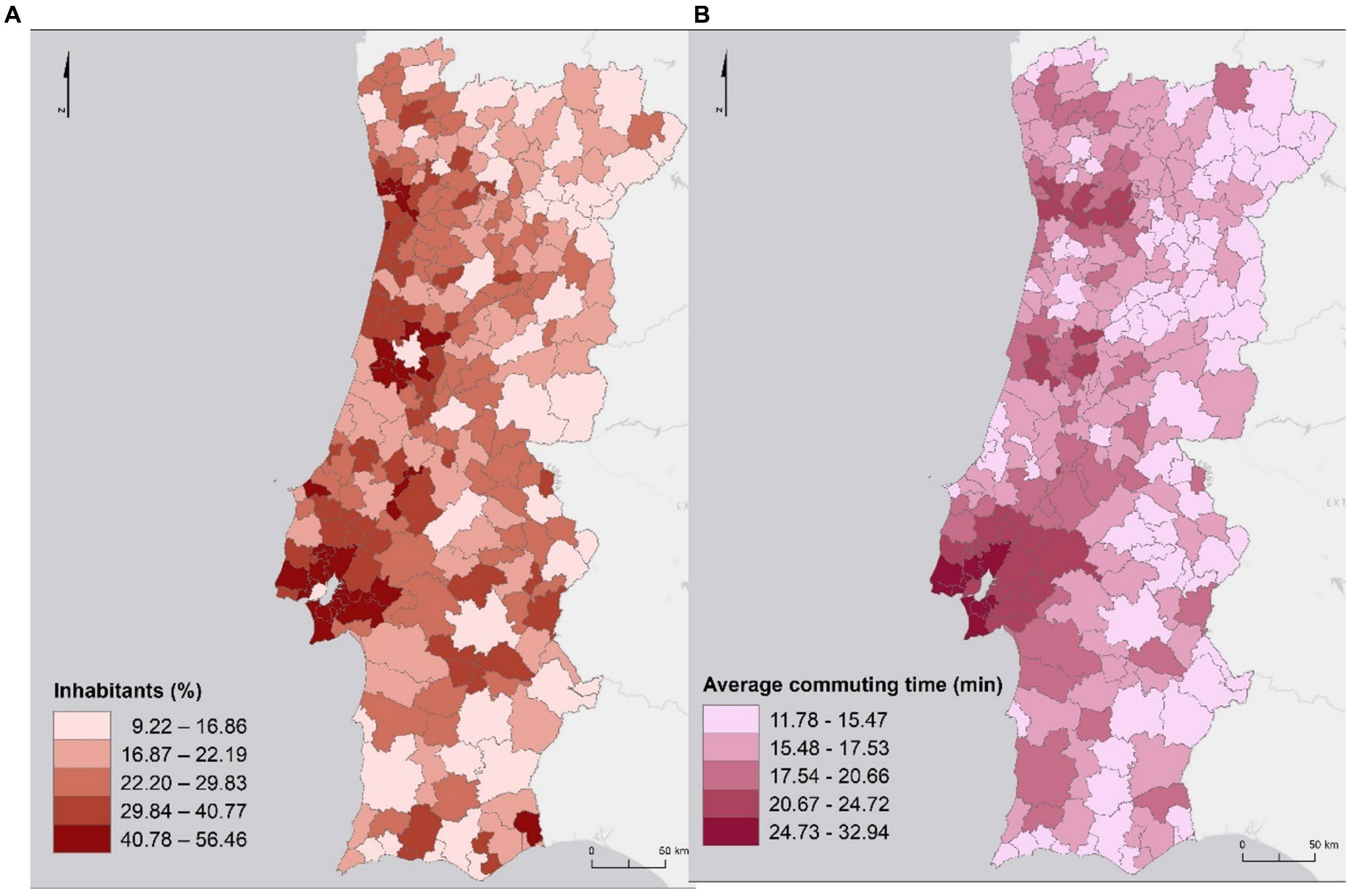
Percentage of inhabitants working outside the municipality **(A)** and average commuting time **(B)**.

Metropolitan Areas (MAs) polarize employment and potentiate commuting since these areas are dominated by a central municipality. In the Lisbon Metropolitan Area (MAL), the difference between central and peripheral municipalities is evident since the neighboring municipalities of Lisbon have higher percentage of inhabitants working outside the municipality. This also stands for Porto in the Metropolitan Area of Porto (MAP), but in a lower extend (Figure 2A). Also, in MAL it can be seen a higher average commuting time (between 26 and 33 min) compared to the peripheral municipalities and the central municipality (Lisbon; between 21 and 25 min). This is because the municipality of Lisbon is the one with the highest job offer. In the municipalities of the MAP, the average commuting time is also higher than the other municipalities (between 21 and 25 min; Figure 2B).

**Figure 3:**
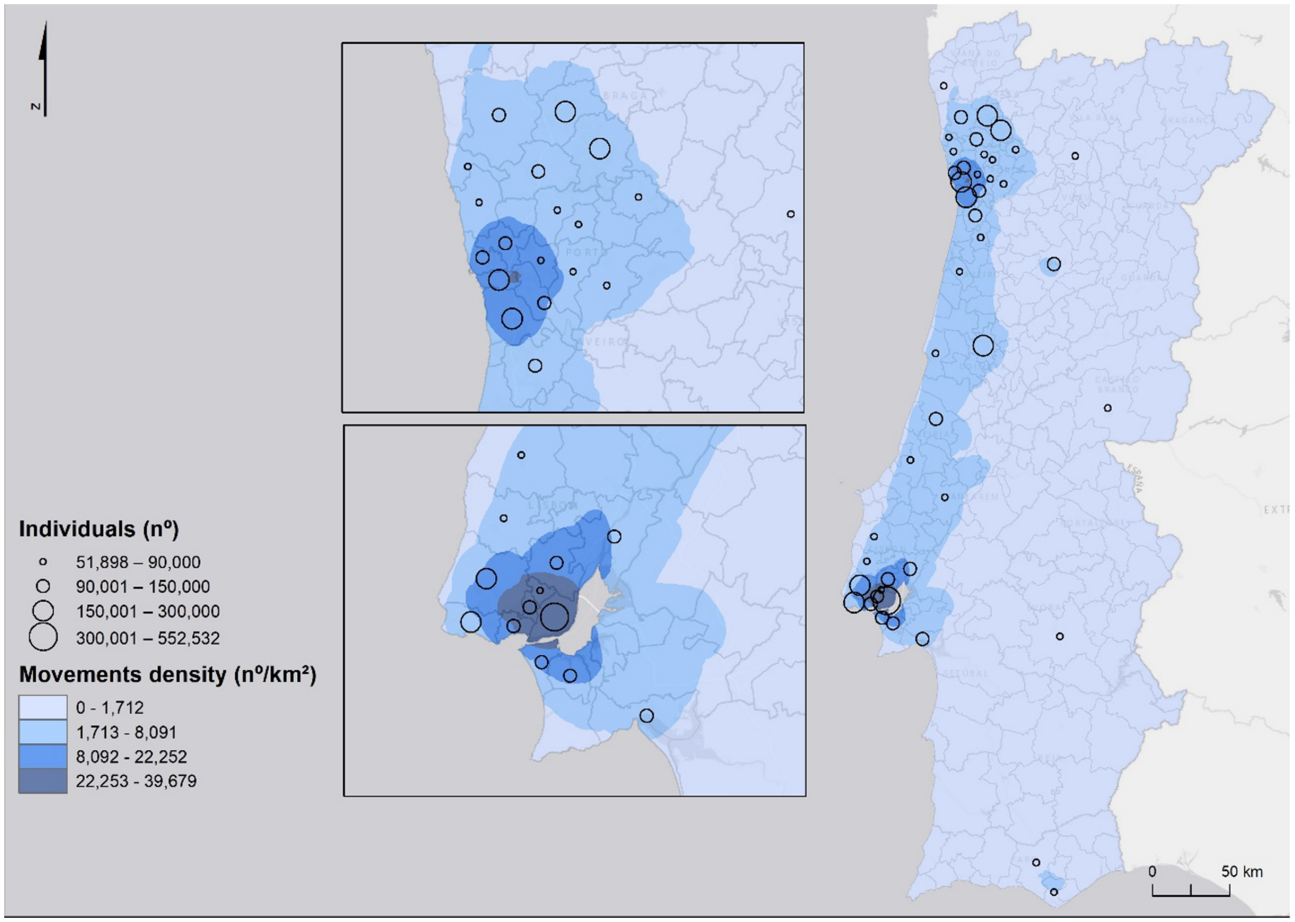
Home work/school commuting.

In general, the average commuting time decreases as the distance to the central municipalities of each of the MA increases (Figure 3).

### 2.2 Model framework

**Figure 4:**
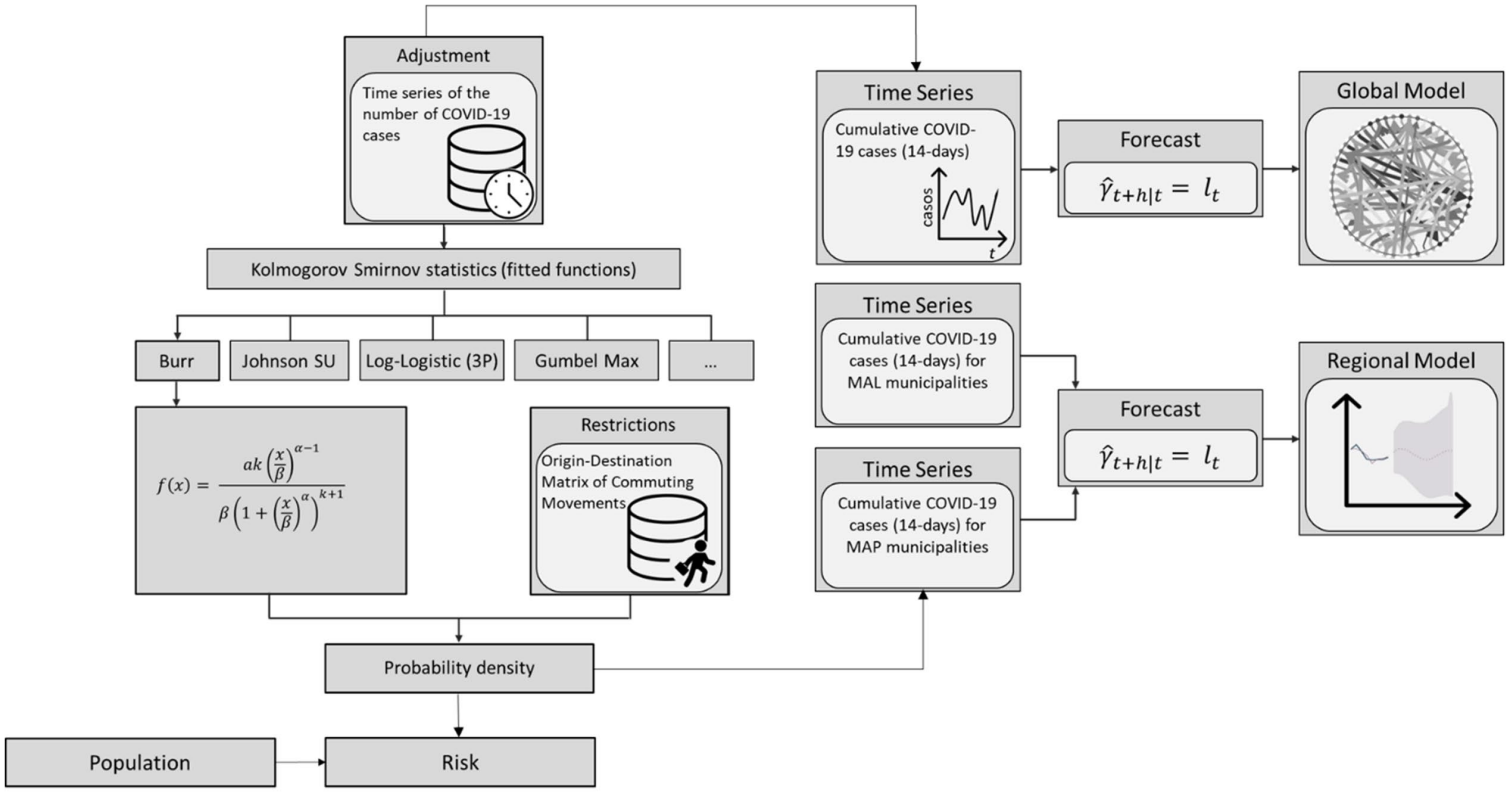
Methodological workflow scheme.

The model framework was developed to identify group of municipalities where population density has a stronger relationship with incidence and commuting movements as well as to estimate the probability of infection and predict short-term evolution of the pandemic at national and regional level. The framework includes five main stages: (1) collection and pre-processing of spatial data, (2) probability density modeling, (3) risk modeling, and (4) global and regional forecasting model. The workflow of the process is shown in Figure 4.

#### 2.2.1 Data

The number of confirmed SARS-CoV-2 infected cases by municipality was collected from July 2020 to July 2022. These data come from the dssg-pt repository, available at https://github.com/dssg-pt/covid19pt-data (36), and sourced from the General Directorate of Health (Direcção Geral de Saúde – DGS) dashboard and the ESRI (Environmental Systems Research Institute) database. We also used mobility data in this study to represent the origin and destination matrix of movements commuting (home-work/school) between municipalities. Due to the importance of movement flows for the transmission of diseases, during the occurrence of epidemic outbreaks, restrictions on movement are among the earliest measures applied, in order to reduce contact between susceptible and infected individuals (37). This data was collected from INE Census 2011, since it was the most recent data made available. It represents the number of individuals who commute daily for work or study between their place of residence (origin) and their workplace or educational institution (destination), categorized by transport mode and sector of activity. It was gathered through an exhaustive inquiry to all the population living permanently in Portugal.

To examine distinct mobility patterns within the country, we combined the data to create different layers: (1) the total number of people traveling between municipalities using any transport mode; (2) the number of people traveling between municipalities using collective transport modes such as train, metro, bus, and boat. Additionally, we gathered statistical data on major sectors of activity based. The sectors of activity are divided into 21 categories. Based on the restrictions imposed by the Portuguese government, we identified the sectors considered essential and not suspended during lockdowns: (i) Human Health and Social Support sector, (ii) the Construction sector, (iii) the Public Administration and Defense sector and (iv) the Students sector.

Moreover, data regarding population density was retrieved for mainland Portugal, on a municipality level, for the year 2020. Data is freely available from the Statistics Portugal (Instituto Nacional de Estatística—INE) portal (38).^1^

#### 2.2.2 Probability density model

We first perform the inferential statistical model to verify which fitted curve best expressed the distribution of the number of SARS-CoV-2 infected cases occurrence as a function of mobility (total commuting movements) for each municipality (39). Therefore, 60 statistics were tested according to the Kolmogorov Smirnov validation method. Our results show that the Burr distribution presents the best fit (Table 1). According to this result, we model the number of SARS-CoV-2 cases by using the density probability equation given by the Burr distribution considering only commuting movements made by Human Health and Social Support sector, Construction sector, Public Administration and Defense sector, and Students sector.

**Table 1:** The 10 best-fitting equations and their rank.

Distribution	Kolmogorov Smirnov	Ranking
Burr	0.04183	1
Johnson SU	0.04508	2
Log-Logistic (3P)	0.04696	3
Gumbel Max	0.04785	4
Pearson 5 (3P)	0.05058	5
Pearson 6 (4P)	0.05059	6
Inv. Gaussian	0.05110	7
Pearson 6	0.05213	8
Lognormal (3P)	0.05265	9
Log-Logistic	0.05330	10

#### 2.2.3 Risk model

The concept of risk in epidemiology designates the probability that an individual in a defined population will develop a disease or other adverse health problem (40). In the context of exposure, vulnerability and risk classifications in the face of an epidemiological phenomenon, it is common to relate the incidence rate in a given period with the exposed population. In this way, a relative risk is obtained that estimates the probability of developing the disease in an exposed population group in relation to another that has not been exposed to the disease. Portugal followed the municipal risk classification according to the European Centre for Disease Prevention and Control (ECDC) categories. Based on the 14-day cumulative incidence values per 100,000 inhabitants, the municipalities are classified according to the risk of infection (< 120 Reduced; 120–239.9 Moderate; 240–479.9 High; 480–959.9 Very high; > 960 Extremely High). Although the concept of risk in epidemiology and public health is conceptually more restricted compared to the concept of risk associated with risk analysis and management (41), which presupposes the combination of different dimensions of risk (exposure, hazard, vulnerability), the classification presented by the DGS is not capable of translating any risk component to SARS-CoV-2 infection. Indeed, the indicator provided by the DGS, which can be considered as an incidence rate weighted by the population, only monitors the epidemiological situation based on transmission, so it should not be considered the only criterion for defining health measures. In addition, the use of the number of inhabitants is highly penalizing for municipalities with a low population. Therefore, we developed a risk classification index used for the definition of containment measures with a view to mitigating the incidence. This index considers how the characteristics of the territories can be aggravating or mitigating transmission, adapting the rigidity of the restrictions in relation to the real danger. In this municipal index of predisposition to infection the most relevant determinants guarantee the worsening or reduction of the index based on the relationship between the determinants and the incidence.

Thus, it is possible to analyze the pandemic waves and cycles for each one of the municipalities and to look for synchronization among them (42). Shaldehi and Dastjerdi (43) calls the attention to the intrinsically ergodicity of synchronized systems. Nonetheless, one not found studies focusing on the pandemic ergodic behavior, and dealing with the commuting movements as a determinant. Ergodicity is a primary notion of physical models that accounts for some degree of randomness in the system. In probability theory, an ergodic system is a stochastic procedure where the average over all its potential states, is the same as its average over time. Therefore, if the expected undulating behavior of the pandemic maintains the same pattern at country our municipality level, recurrently over time and in specifically regarding the peaks, the required circumstances for the pandemic periodicity can be described through the ergodicity concept. However, geographical neighboring does not ensure commuting movements between municipalities (44, 45).

Because the sample function of the number of cases is assumed to follow a Burr distribution, where the shape factor k represents the totality of all determining factors (including the mobility factor); and the approximation of one sample wave of the SARS-CoV-2 pandemic, to the Burr distribution is clearly observable from our calculations, we used a mobility factor for the purpose of integrating commuting movements in risk analysis. This mobility factor (M) is assumed to have an exponential propensity toward k . The rational is that the infection evolution degree is connected to the Burr distribution shape factor and M need to be linked to k for adjusting to the prediction model. This mathematical model undertakes an exponential relationship between k and M to highpoint its significance, $M=re^{\beta k}$ where β is the scale factor.

Since all states are recurrent and aperiodic (46) M can be characterized as a matrix, representing the commuting movements between all the municipalities. The ones with high SARS-CoV-2 incidence are committed by a supplementary factor (1+q) , where q stands for the fuzzy weight of incidence of each municipality within Portugal mainland context; for municipalities with no cases (q=0) . The commuting between distinct municipalities can then be recognized as $M_{ij}^{1}$ , $M_{ij}^{2}$ , …, $M_{ij}^{n}$ , supposing a normalized element in a fuzzy system $0\leq M_{ij}^{n}\leq 1$ , where i is the population moving to the municipality and j the population moving from the municipality.

The net value of M for the municipality (i) can be computed by means of the arithmetic average of the product of (1+q) and the interaction $M_{ij}^{n}$ :


$$M_{i}^{n}=\left(\frac{1}{n-1}\right)\overset{n}{\underset{j=1}{\sum }}\left(1+q\right)M_{ij}^{n}$$

#### 2.2.4 Global and regional forecast model

As the spread of infectious diseases is a mixed process of random and individual nature (47), the description of transmission between individuals is better adjusted using probabilistic models (48). The behavior of an infectious disease in a population reflects an average of all infected cases. Although the impact of each infected individual on the outbreak is unpredictable, the overall population dynamics typically align with mathematical predictions. This is because random variations among individuals tend to balance out as the number of infected people grows, following the law of large numbers (49). Directed and weighted human movement networks, integrating human flow intensity with neighborhood diversity metrics can help pinpointing super-spreader and super-susceptible locations (50). As the number of individuals in the network increases, the law of large numbers helps to ensure that these identified locations reliably reflect the true dynamics of disease spread within the population. Hence, even if the distribution of secondary cases is highly uneven, the epidemic will generally progress smoothly as long as the expected number of new cases per observation period is sufficiently large. Conversely, if the infection rate is low, the epidemic may exhibit more complex and fluctuating dynamics (49).

Stochastic approaches are particularly suitable when the population under study is small. These models consider the extinction of a disease to be possible in a finite time, unlike deterministic models that tend toward a situation of endemic equilibrium (51). Therefore, we applied the Simple Exponential Smoothing (SES) to estimate the prediction values for a future period. Particularly, we chose this model as it was deemed most appropriate for data that does not follow a clear trend or seasonality pattern. This predictive model consists of an algorithm that comprises a stochastic data generation process, which can be used to produce the entire probability distribution for a future time interval n+h . Thus, a point forecast is obtained via the mean (or median) of the probability distribution. This model also allows the calculation of forecast intervals with a given confidence level. The predictions are weighted averages of past observations. The term exponential smoothing assumes the fact that the weights decrease exponentially as the observations are older (52), i.e., the more recent the observation the more importance is given to the value. Thus, by applying the weighted average, all predictions for the future are equivalent to the simple average of the observed series (53–56):


$${\hat{y}}_{T+h|T}=\frac{1}{T}\overset{T}{\underset{t=1}{\sum }}y_{t},$$

for h=1,2,… . Thus, the averaging method assumes that all observations are of equal importance and assign equal weights when generating future predictions. The predictions are calculated based on weighted averages, in which the weights decrease exponentially as observations come from the oldest records. The smallest weights are associated with the oldest observations:


$${\hat{y}}_{T+1|T}=\alpha y_{T}+\alpha \left(1-\alpha \right)y_{T-1}+\alpha {\left(1-\alpha \right)}^{2}y_{T-2}+\ldots ,$$

where 0≤α≤1 is the smoothing parameter; the “one step forward” prediction for time T+1 is the weighted average of all observations in the series $y_{1},...,y_{T}$ ; and the speed at which the weights decrease is controlled by the parameter α .

The application of any exponential smoothing method requires that the smoothing parameters and initial values be set. In particular for SES, for which the values of α and $l_{0}$ are selected. All predictions can be computed from the data since we know these values. For the following methods, it is usual to have more than one smoothing parameter and more than one initial component to be chosen (56).

In some cases, smoothing parameters must be chosen subjectively - for example, based on the value of previously tested smoothing parameters. Nevertheless, a more trusted and objective way to obtain unknown parameter values is to estimate them from observed data. Thus, the unknown parameters and initial values can be estimated by minimizing the sum of squared errors (SEE). The residuals are specified by $e_{t}=y_{t}-{\hat{y}}_{t|t-1}$ for t=1,…,T . Therefore, the values for the unknown parameters and the initial values that minimize are found (56):


$$SEE=\overset{T}{\underset{t=1}{\sum }}{\left(y_{t}-{\hat{y}}_{t|t-1}\right)}^{2}=\overset{T}{\underset{t=1}{\sum }}e_{t}^{2}\;$$

The GM was first evaluated through the coefficient of determination $\left(r^{2}\right)$ that evaluates the dispersion of the data points around the fitted regression line. The $r^{2}$ is applied in computing the explaining capability of the independent variables in the model. This varies between 0 and 1, ant the higher the values, the smaller the difference between the observed data and the predicted values and, therefore, the better the model. Complementarily, Root Mean Squared Error (RMSE) was used to calculated the root mean squared of the errors between observed (actual) values and predictions (hypotheses). Both RMSE and $r^{2}$ allows to quantifies the level of agreement (i.e., fitness) between the model and the dataset. However, the RMSE gives the effectiveness of the model in predict the response variable in absolute values while $r^{2}$ shows how good are the explaining capabilities of the predictor variables regarding the changes in the response variable.

A feature of RMSE is that errors (actual–predictions) are squared before being averaged. Therefore, different weights will be assigned to the sum and, as the error values of the instances increase, the RMSE increases accordingly. If there is an outlier in the dataset, its weight will be high within RMSE calculation and, as a result, it will affect the metric. This can be problematic when comparing RMSE results calculated on test samples of different sizes, which is often the case in real case modeling, i.e., this metric alone may not describe the mean error. To avoid this problem we applied the Akaike Information Criterion (AIC) to evaluate the goodness-of-fit of the model (57). This method is based on the concept of entropy, i.e., information theory, and measure the loss of information when a given model is used to define reality. It can be described as the exchange between bias and variance in the construction of the model, or simplifying, between the accuracy and complexity of the model. The bias is asymptotically given by k, where k is the number of parameters to be estimated in the model (if the estimated model is a linear regression, k is the number of regressors, including the intercept), and defined its information criterion as:


AIC=−2ln[f(x|∅)]+2k,

where x is the observed data, ∅ is the maximum likelihood estimation, i.e., f(x|∅) is the probability of the observed data due to the number of parameters k or the likelihood of the parameters given the dataset, and ln is the natural logarithm.

Overall, the SES model was applied to all municipalities in Continental Portugal, obtaining a Global Model (GM), and also for the municipalities of MAL and MAP, making a prediction for the future of 1 year, obtaining a Regional Model (RM). However, the RM was evaluated using the F−statistic that for an independent variable with k groups evaluates whether the group means are meaningfully different. The F−statisti c is computed as:


$$F=\frac{MSR}{MSE},$$

where MSR is the regression mean square and MSE the mean square error. To considerer the F−value one need to look at p−value . If the latter is low (i.e., lower than the α−level we can reject the null hypothesis and accept the model as valid) (58). When the *F*-value is below α, then the null hypothesis is true, i.e., when it presents very high values, then the variation between the group averages is greater than expected and the variation within the group is greater than a random variation. The high values of F may mean that the null hypothesis is not true (the data are not sampled from populations with the same mean), or that the random sample has large values in some groups and small values in other groups, which seems to corroborate the results of the spatiotemporal analysis where only oscillating patterns were found.

## 3 Results

### 3.1 Probability density and risk model

**Figure 5:**
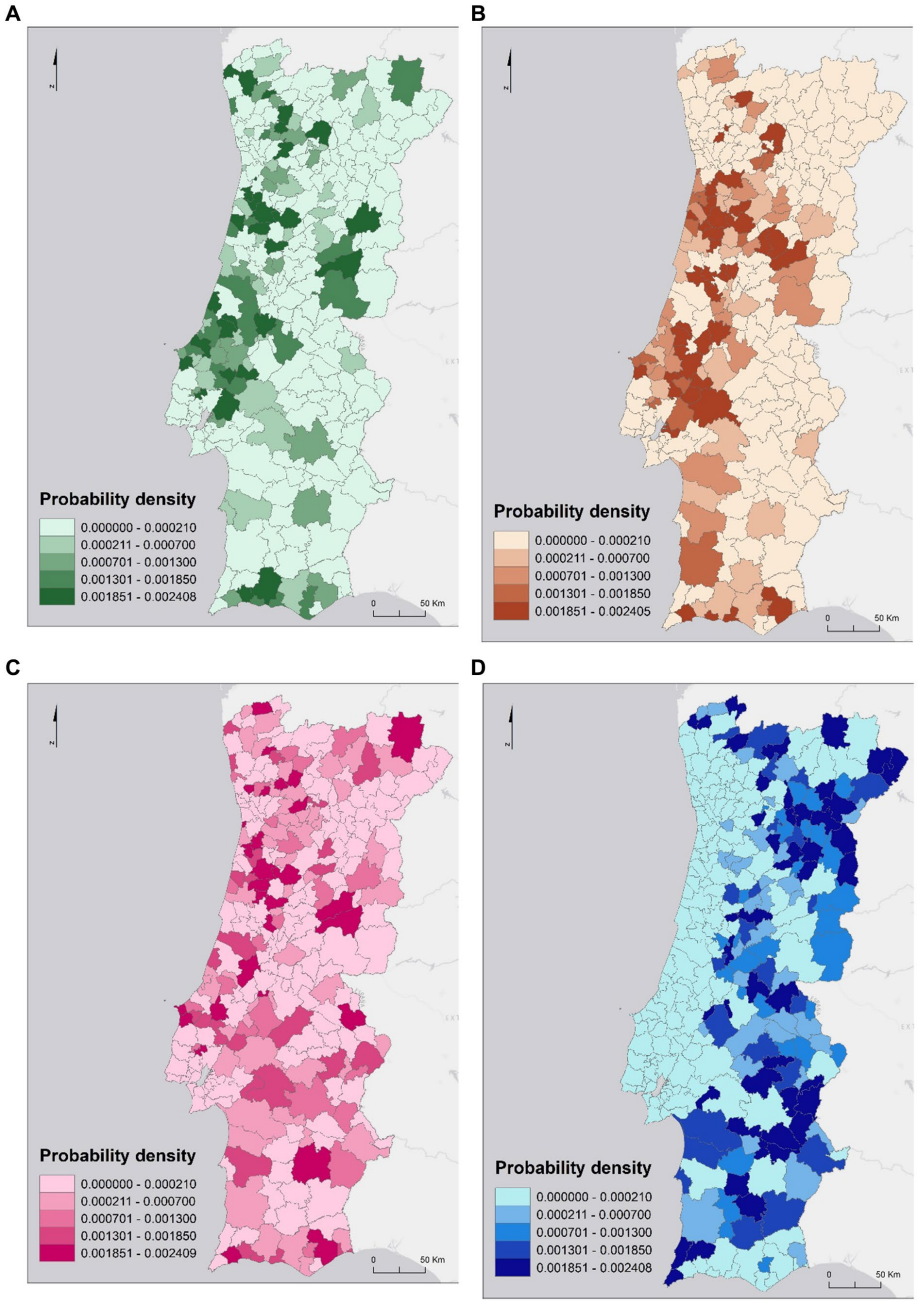
Density probability of the number of SARS-CoV-2 infected cases by commuting movements in the human health and social support sector **(A)**, the construction sector **(B)**, the public administration and defense sector **(C)** and the students sector **(D)**.

When modeling the density probability of SARS-CoV-2 infected cases as a function of mobility for certain economic activities groups a similar spatial distribution pattern is found for Human Health and Social Support, Construction and Public Administration, and Defense, and a different pattern for Students (Figure 5). If only workers of Human Health and Social Support move (Figure 5A), the density probability of case is higher (above 0.001851) in municipalities on the North and Central coast, in the Algarve, and also in some municipalities in the Central interior. If only individuals working in the Construction sector commute (Figure 5B), the municipalities with a higher density probability of SARS-CoV-2 infected individuals are mostly in the Central region, northwestern Alentejo region, and Algarve. The coastal strip from Mira to Caldas da Rainha presents lower values. As for AMP and AML, there was only one municipality with high values in each, namely Espinho and Alcochete, respectively. If only individuals employed in the Public Administration and Defense made commuting movements, then the density probability of SARS-CoV-2 incidence would be higher in some municipalities in the North (e.g., Monção, Bragança, Amares); Center (e.g., Águeda, Tondela, Anadia; Peniche); Alentejo (e.g., Portalegre; Beja) and Algarve (e.g., Lagos and Tavira) regions. In AMP only the municipality of Trofa presents a higher probability and in AML all municipalities present a very low probability (less than 0.000210).

If only students are commuting (Figure 5C), then the spatial pattern of spread of the density probability of SARS-CoV-2 infection cases is different from the other sectors under analysis, with a generally higher probability in inland municipalities. In this sense, municipalities with a higher density probability are mostly from the eastern region of Terras de Trás os Montes (e.g., Vimioso and Miranda do Douro), Douro (e.g., Torre de Moncorvo, Vila Nova de Foz Côa), Beiras and Serra da Estrela (e.g., Figueira de Castelo Rodrigo, Trancoso) and Alentejo Central (e.g., Alandroal, Redondo), and the Western region of Baixo Alentejo (e.g., Aljustrel, Castro Verde) and Algarve (e.g., Aljezur and Vila do Bispo). If only students were commuting (Figure 5D), the spread patterns could be quite different from the others since there are many educational institutions in the inland municipalities to which commuting takes place.

**Figure 6:**
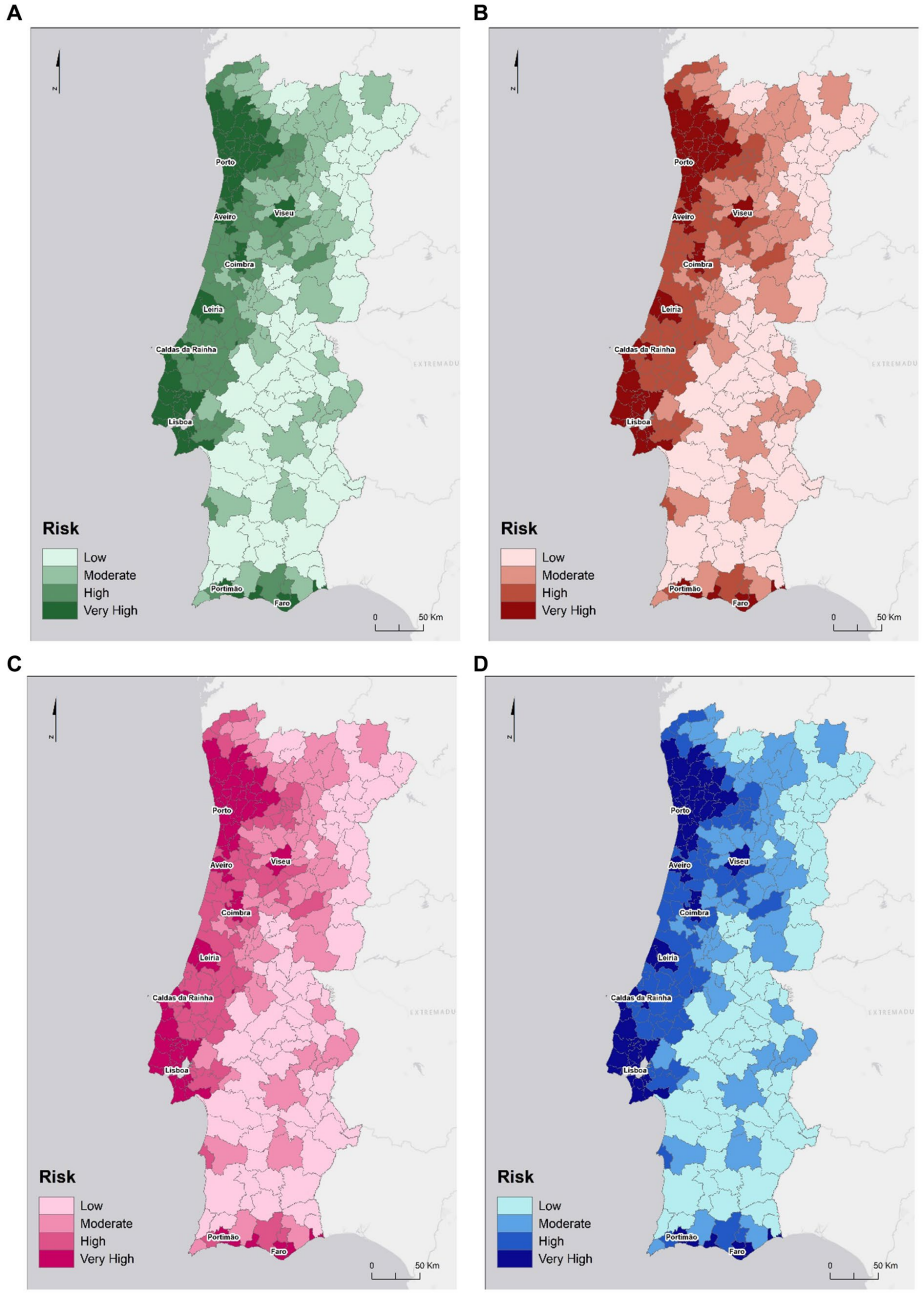
Risk of SARS-CoV-2 infections considering existing cases, population density and commuting movements in the human health and social support sector **(A)**, the construction sector **(B)**, the public administration and defense sector **(C)** and the students sector **(D)**.

The classification of municipalities based on the risk interpretation shows that the most important group for all sectors were both metropolitan areas and surrounding municipalities (Figure 6). The second group, the density highlights most of the remaining territory within the functional dependencies of metropolitan areas (not so much in the case of MAL), where spatial contiguity and adjacency play an important role, and relevant municipalities in the national urban hierarchy, such as district capitals (Coimbra and Aveiro), with a high economic dynamic (Leiria and Caldas da Rainha) and some important cities in the Algarve region (Faro and Portimão). The remaining group, concerns the majority of the municipalities in the interior, especially the ones near the border with small size and/or low population density. The risk information shows that higher density territorial units present more probability of facing intense and prolonged outbreaks, as opposed to less dense areas with rural characteristics.

### 3.2 Forecast model

**Figure 7:**
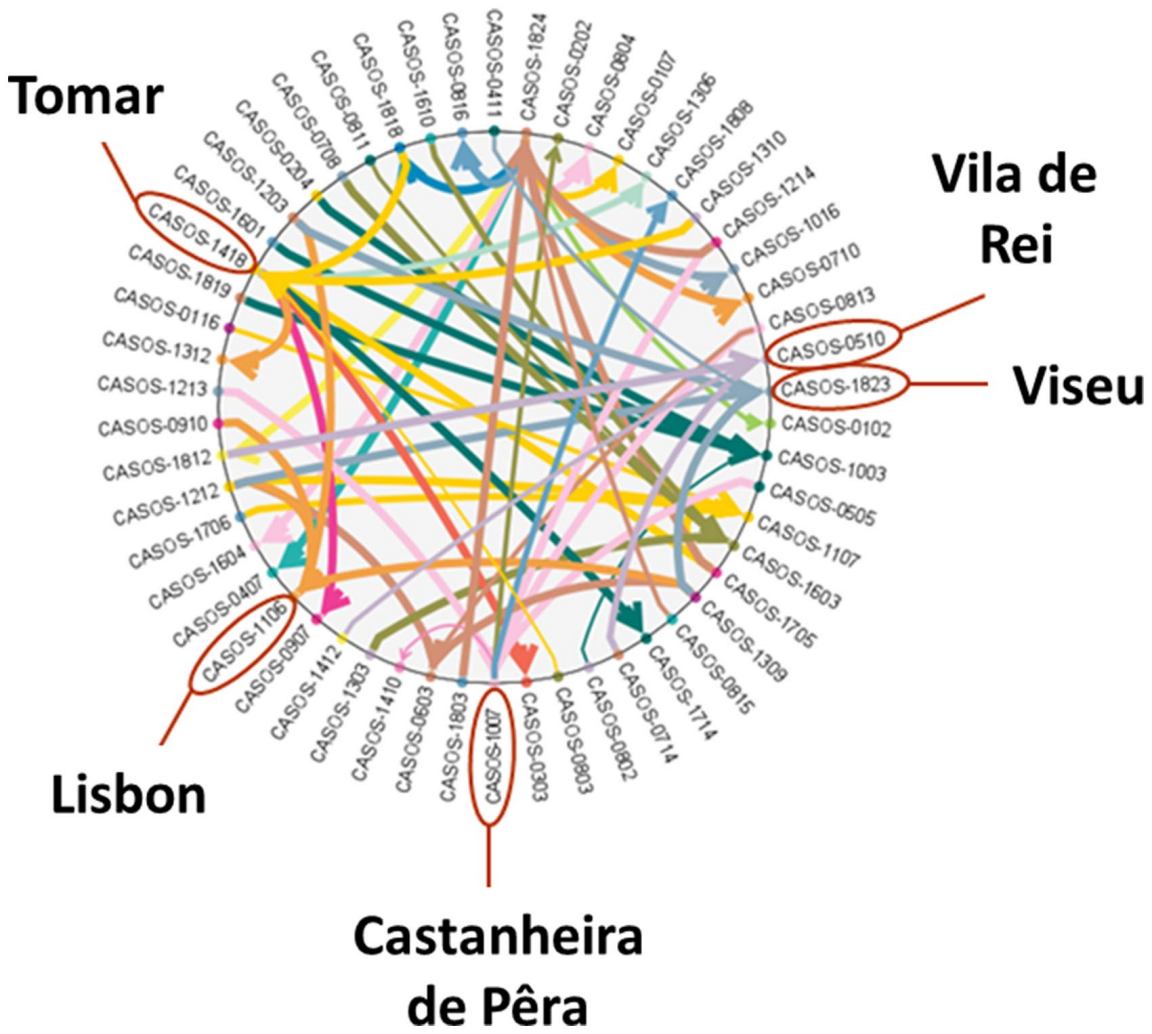
Global model scheme.

In the GM scheme, it can be seen that the municipalities most related to each other are Viseu (1823) and Lisbon (1106) and, on the opposite, Castanheira de Pêra (1007), Tomar (1418), and Vila de Rei (0510; Figure 7). The coefficient of determination of the global predictive model reflects a good fit since it varies between 0.88 and 1 for all municipalities. As for the RMSE, it can be seen that most of the occurrences have an RMSE close to 0, which suggests a good predictive ability. Regarding the AIC of the predictive model, this was the one with the lowest county-by-county values compared to others that were run, with most observations being between 56.38 and 225.5. The fit for each municipality follows a normal distribution.

**Figure 8:**
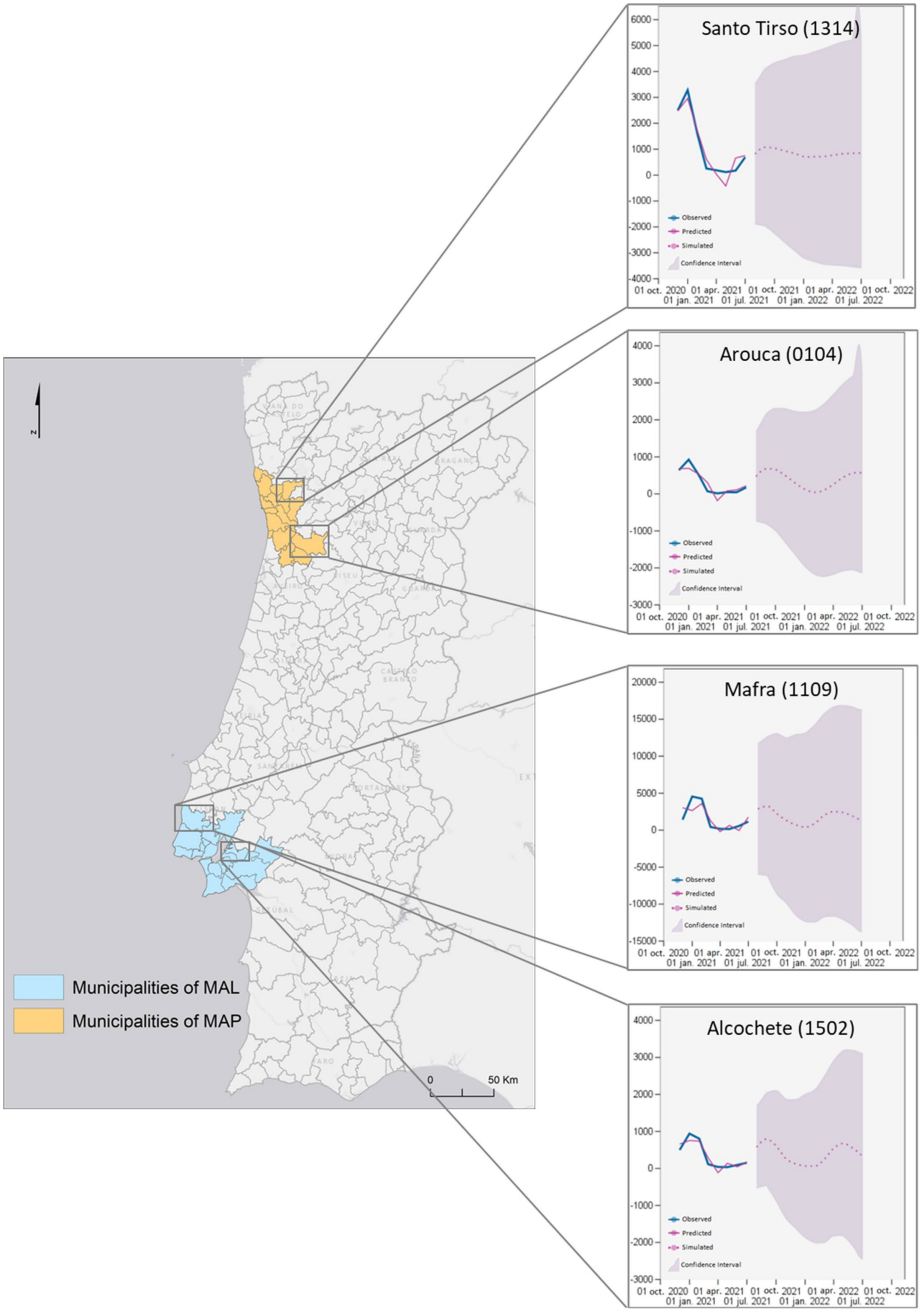
Regional model forecast for two municipalities of MAP and two of MAL.

Figure 8 presents the results of the RMSE prediction performed for the municipalities of MAL and MAP, from which two municipalities were selected for each of the MAs. Predicted results refer to outcomes that are computed based on the model and then compared to actual events. Simulated results represent the expected future behavior of the system based on current knowledge and assumptions. These results are used to test hypotheses or explore scenarios that are difficult to study directly.

All predictions take the same value as in the last level component. The selected municipalities presented values of r2 equal to 1, i.e., they present a complete adjustment to the variation in the data. According to the prediction made for the municipalities of MAP, the behavior of the prediction curve for the municipality of Santo Tirso suggests that the number of cases of SARS-CoV-2 infection may range from 0 to 1,000 cases accumulated at 14 days per 10 thousand inhabitants. Reaching the maximum prediction value in October 2021. For this, the *F* value is high, around 4.89 (α=1.4673) . However, this prediction presents a high *p*-value. The municipality of Arouca also presents an oscillation movement of the predictive values, as far as the prediction is concerned, between 0 and 800 accumulated cases at 14 days per 10 thousand inhabitants, and it is expected to reach the maximum value in October 2021, and the minimum value in February 2022, rising again in July 2022. This municipality presented an F value of 1.72.

Compliant with the prediction made for AML municipalities, it is estimated that, in Mafra, the number of cases of SARS-CoV-2 infection will vary between 0 and 3 thousand cases accumulated at 14 days per 10 thousand inhabitants. It reaches its maximum value at the end of September 2021, the minimum in January 2022, rising again to approximately 2,500 cases in April 2022. This prediction has an F value close to 1 (0.67). The municipality of Alcochete also presents an oscillation of the predictive values, suggesting that the values vary from 0 to approximately 900, reaching the maximum in late September 2021, the minimum during January and early February, rising again to approximately 800 in May 2022. The *F*-value for the prediction for this municipality is 2.41. In general, according to the predictive model applied, the number of accumulated 14-day cases of SARS-CoV-2 infection is expected to oscillate in all municipalities, reaching higher relative maximum values in Mafra (3,000 accumulated 14-day cases of SARS-CoV-2 infection per 10 thousand inhabitants) and lower values in Arouca (800 accumulated 14-day cases of SARS-CoV-2 infection per 10 thousand inhabitants). All estimated values remain in the same range of values of the observed values.

According to the law of large numbers, as the number of individuals in the network increases, the aggregated data ensures that these identified locations reliably reflect the true dynamics of disease spread, thereby aligning both the predicted results and the simulated results with actual epidemic patterns.

## 4 Discussion

Restrictions and limitations on population displacement are one of the main non-therapeutic intervention measures adopted in epidemic contexts (59, 60). During the evaluation of the SARS-CoV-2 pandemic several barriers to the free movement of the population were adopted, both internally and internationally. The objective was to promote social distancing and isolation (61), due to the existence of evidence that social distance resulting from the application of mobility restrictions reduces the virus transmission (62). Jia et al. (63) were among the first to associate population movements with the spatiotemporal distribution in China. The authors demonstrated how data on population mobility flows explain the spatiotemporal evolution of incidence. In fact, mobility is an important factor at multi-scale, i.e., from local to global, with connectivity between locations constituting an explanatory factor for the territorial variation of infections (64, 65). Thus, population displacement has been identified as a determinant for the spread of SARS-CoV-2 since the beginning of the pandemic.

Our results reinforce the conclusions of several studies (66, 67) that stressed the existence of a relationship between mobility and the number of infections, showing the relevance of considering mobility indicators in epidemiological modeling of SARS-CoV-2, although with temporal and spatial variations. The value of the mobility factor can be allocated after considering various interactions between municipalities. Once a municipality assumes an ergodic behavior the majority of its population becomes infected. The possible inexistence of preventing or mitigation measures can turn the scenario even worse. Moreover, due to its high transferring rate SARS-CoV-2 carries a high probability for random (perhaps chaotic) outbreaks and faster spatial diffusion.

In the specific case of mobility, the use of “real-time” data, for example based on the geolocation of mobile phones, would be very useful in the event of an epidemic, as it makes it possible to assess, at any time, the degree of mobility of the population and its geographic patterns (68, 69). Another relevant aspect of mobility is the movement, and presence, of a large volume of individuals associated with certain events (super-spreading events) that increase the possibilities of contagion to an outbreak scale (70). However, in this case the incorporation into the modeling processes is more difficult because it underlies a dimension for which it is difficult to obtain reliable information.

Numerous simulation models argue the basis of pandemics spreading behavior as single peaked, i.e., without considering the probability of an episodic recurrence. Increased commuting movements’ increases the prospective of occurring random outbreaks and, thus, the spreading of the virus. Once the municipalities ease movement restrictions, social distancing or lockdown measures, the odds of a new outbreak in the same administrative region escalates. International mobility and the increasing flows of interaction associated with globalization, by reducing epidemiological differences between regions (71), constitute a difficulty in the modeling of infectious diseases that has to consider how the flows of population movements can be important covariates, for the prediction of future epidemic evolution. Thus, epidemiological models must have a spatial dimension to predict the spatiotemporal spread of pathogens.

The metapopulation version of dynamical deterministic/stochastic mathematical models (72) and the Simple Exponential Smoothing (SES) model offer complementary approaches to understanding and predicting epidemic dynamics. Metapopulation models, which account for multiple interacting subpopulations, provide a detailed representation of disease spread, incorporating spatial heterogeneity and local variations in transmission dynamics. These models can handle complex scenarios, such as varying infection rates and mobility patterns, and can simulate both deterministic and stochastic effects, making them powerful tools for exploring different intervention strategies and understanding epidemic behavior at a coarse level (73). On the other hand, the Simple Exponential Smoothing model is a time series forecasting method that excels in capturing short-term trends and smoothing out random fluctuations in data. It is particularly useful for producing quick, computationally efficient forecasts based on recent data, without the need for detailed knowledge of the underlying transmission mechanisms. SES is ideal for situations where rapid predictions are needed, and it works well with stable data that follows an exponential trend. By leveraging both approaches, researchers and public health officials can gain a comprehensive understanding of epidemic dynamics, combining detailed spatial and stochastic insights with efficient short-term predictions.

In an epidemic context, it is essential to implement spatiotemporal surveillance systems that prioritize interventions in areas at high risk of infection (67). Likewise, the territory can be classified by its predisposition to the transmission of the virus, according to the known relationship with the determinants of infection. The idea of classifying the territory to the danger of contagion results from the need to delineate spatial-based Public Health measures. Nonetheless, these measures should consider the characteristics of the territories on which they affect because what works well in one set of circumstances may be inappropriate for another, therefore, universal panaceas are not recommended (66), i.e., one size does not fit all. Our results regarding the risk index shows that higher density territorial units present more probability of facing intense and prolonged outbreaks, as opposed to less dense areas with rural characteristics. This finding is in line with the epidemiological theories presented before (74). Living in higher density places implies a greater probability of social contact and generally corresponds to areas with an aging population and where income disparities are higher (75). In metropolitan contexts, the structured connections in the transport networks, in economic terms and in terms of commuting, are pointed out as explaining the central-periphery patterns of infections (76), namely when these movements are carried out via public transport, for example. Greater exposure to contagion (25). Similar, Mourao and Bento (77) identified the relationship between the contiguity between territorial units and the progression of pandemic spread in Portugal, highlighting the importance of mobility between municipalities for contagion, in line with the conclusions of Silva et al. (45) and Oliveira et al. (44).

Networking models prove the validity of traditional spatial diffusion models, but also helps in the interpretation of complex patterns, based on vast ties of social relationships, of which spatial diffusion models are not representative. In a process of infectious disease spreading having knowledge of the networks of social relationships of individuals is crucial to identify suspected cases and determine the full extent of the transmission chains (27). In the context of research into the relationship between SARS-CoV-2 and its determining factors, the adoption of global models continues to be dominant (78). At this stage, there is the advantage of using spatial regression models by incorporating the importance of distance for the dependent variable, similar to others applied to the context of SARS-CoV-2 (79–81), determining the spatial variability of the relationship between incidence and factors previously identified as most relevant and significant for disease patterns in mainland Portugal. This is particularly evident when mixed diffusion processes whose intensity and importance vary in space and time are manifested. HIV-AIDS is an example of a pandemic phenomenon whose spread is shown to result from the combination of expansion (contagion and hierarchical) and relocation processes (82). The occurrence of these processes derives from different forms of transmission, but whose diffusion increases with more exposure, i.e., the denser the social networks of the individuals (29).

Regression models are also commonly used to measure the relationship between the prevalence of a disease in a given context and possible risk factors - a set of independent explanatory variables (5). Based on the quantification of this relationship, it is also possible to predict future developments both in time and in space. Regressions can assume different formulations depending on the type of relationship expressed between the epidemic process and the explanatory factors (83). Nevertheless, the behavior of a pandemic is somehow chaotic, due to the difficulty in extracting trends that can be applied to other contexts and scales of analysis. The different waves of the disease are conditioned by the occurrence of those that precede them (84), but as there is no symmetry between them, nor a fixed period, i.e., their future occurrence entails greater forecast risks. The statistical complexity of the phenomenon is compounded by the fact that the permanence of the disease in a territory has never shown to be static. For instance, predictions are only suitable for time series without the trend and seasonality components (53–56). SES model gives more importance to recent occurrences than the latter ones, transposing to the GIS fourth dimension, i.e., time, the spatial first law of geography, that everything is related but near things are probably more related (85).

Brinks and Ibert (86) indicated the need to integrate the spatial component in conceptualizing the risks associated with the pandemic, as Mourao and Bento (77) and Marques (66) reinforced the importance of adopting public health measures aimed at the specificities of the territories. This is because the unequal incidence of SARS-CoV-2 results from different levels of vulnerability of municipalities to infection, resulting from areas such as external exposure, population of the territory, sociodemography, interaction through mobility and exposure through occupation (31, 44, 66, 87), then it is essential that containment policies consider these differences. Nevertheless, the selection of disease transmission models for a given approach under study must be the result of a trade-off between complexity and precision. An adequate model for the study of a given pathology must result from what is intended to be achieved, in a compromise between simplicity, omitting details and demonstrating global qualitative patterns, and the detail, for specific situations and quantitative predictions (88).

Overall, SES requires relatively fewer data points compared to more complex models. It can generate reliable short-term forecasts even with limited or infrequent epidemiological data, making it suitable when data is not consistently available. For example, despite it does not explicitly incorporate sociodemographic or environmental factors, it can effectively capture and smooth trends in case numbers, reflecting the aggregate impact of these factors without needing detailed, granular data. It inherently smooths out random fluctuations and noise, which helps in managing non-linearity and temporal dependencies without the need for complex adjustments. Therefore, provides a straightforward approach to capturing the overall trend, which can be particularly useful when more sophisticated methods are hindered by data limitations.

The proposed method focuses on observed trends and recent data, which can help mitigate the impact of inconsistencies in testing rates and reporting quality (e.g., can smooth out the noise caused by reporting delays, giving a better picture of the global trend). By giving more weight to recent observations, it makes it easier to detect changes in the epidemic’s trajectory despite reporting inconsistencies and can adapt to changes in testing policies and practices more dynamically than more complex models that might require a stable testing regime. Finally, while SES does not directly address ergodicity, its simplicity and focus on recent trends can help reduce some biases caused by data quality issues and reporting delays, offering more reliable short-term forecasts compared to more complex but data-sensitive models.

## 5 Conclusion

Density functions for data fitting must be selected with extreme precision, particularly in situations such as pandemic outbreaks. Simple linear or logistic regressions are not suitable for this type of data due to autocorrelation and the influence of an excessive number of zeros. Our tested models provide a good alternative for when explanatory data is few and the time component is not available, once they have shown a good fit and good short-term forecast ability. The effective use of time is of utmost importance for the study of phenomena such as the SARS-CoV-2 pandemic since it enables the interpretation of the event over time for the area under study. The patterns of virus spread would be similar if only people from the Human Health and Social Support, Construction and Public Administration, and Defense sectors commuted. However, if only students were commuting, the pattern of distribution would be quite different and more concentrated in the interior, since there are many educational institutions in the country’s inland municipalities.

We can state that human mobility and transportation patterns are associated with the dissemination of diseases in the population. The patterns of virus dissemination vary between the continental territories and especially, in metropolitan areas and between their municipalities, indicating different mobility dynamics, which should be considered in health strategies and public policies. Over time the tendency is that the number of cases will continue to oscillate, one of the reasons being the application and readjustment of the restriction measures adopted by decision-makers. In this context, mathematical modeling of infectious diseases can provide important insight into the stage of an epidemic and its evolution. Together, these models provide a robust toolkit. For instance, metapopulation models can offer in-depth insights and scenario analysis, while the SES model can deliver quick, reliable forecasts. By leveraging both approaches, researchers and public health officials can gain a comprehensive understanding of epidemic dynamics, combining detailed spatial and stochastic insights with efficient short-term predictions.

Despite the results we have achieved, some limitations were identified, namely the lack of data that could be integrated into the predictive model as explanatory variables (e.g., mobility) since they are not available for time intervals. Another limitation was the fact that data on cases of SARS-CoV-2 infection is available at the municipal level and aggregated in periods of 14 days, which does not reflect the daily reality of the number of cases, making a more detailed analysis impossible.

Further research can be used to study SARS-CoV-2 in a GIS environment, e.g., daily mobility data from social networks or mobile networks, to enable the integration in the predictive model. In addition, it would also be interesting to apply a multi-agent system to model the possible future behavior of the virus dispersion. Through this methodology, the agents reproduce an autonomous behavior (through decision making) and interact with each other through the definition of some functionalities (coordination, cooperation, competition, and negotiation), enabling machine learning. In this way it would be possible to insert limiting factors (such as restrictions on mobility) and predict the repercussions these constraints would have.

In short, we can argue that SES is not a replacement for more detailed and comprehensive models, it offers a practical and robust alternative for short-term forecasting, especially in contexts where data is incomplete, inconsistent, or delayed. Its simplicity and adaptability make it a valuable tool in the context of epidemiological modeling, providing quick and reliable insights that can inform public health decisions almost in real-time.

## Data availability statement

Publicly available datasets were analyzed in this study. This data can be found at: https://github.com/dssg-pt/covid19pt-data.

## Author contributions

MS: Conceptualization, Data curation, Formal analysis, Investigation, Methodology, Validation, Writing – original draft. CV: Data curation, Formal analysis, Methodology, Validation, Writing – original draft. IB: Data curation, Formal analysis, Writing – original draft. PN: Methodology, Supervision, Validation, Writing – review & editing. RR: Supervision, Validation, Writing – review & editing. JR: Conceptualization, Funding acquisition, Supervision, Validation, Writing – review & editing.

## Footnotes

^1^ https://www.ine.pt/
